# Pulsed magnetic field increases the effect of ultraviolet C radiation and thermal shock in aged yeasts

**DOI:** 10.1007/s10123-023-00352-2

**Published:** 2023-03-30

**Authors:** Silvia Mercado-Sáenz, Alejandro González-Vidal, Antonio M. Burgos-Molina, Beatriz López-Díaz, Francisco Sendra-Portero, Miguel J. Ruiz-Gómez

**Affiliations:** Laboratorio de Radiobiología, Departamento de Radiología y Medicina Física, Facultad de Medicina, Universidad de Málaga, Bulevar Louis Pasteur, 32, 29010 Málaga, Spain

**Keywords:** Magnetic field, heat shock, ultraviolet C, radiation, yeast, *Saccharomyces cerevisiae*

## Abstract

The study of the effects of the magnetic field (MF) on living matter continues to be a dilemma. Until now, the interaction mechanisms of MF with living matter that explain the observed phenomena are unknown. Despite the existing literature and the multiple effects described to date, there are few published articles that study the combined effect of MF with other physical agents during the cellular aging process. In this sense, the aim of this work is to study whether low frequency and intensity pulsed and sinusoidal MF exposure produce alterations in the cell killing effect of ultraviolet C (UVC) radiation and thermal shock during the chronological aging of *S. cerevisiae*. Yeast cells were exposed to 2.45 mT (50 Hz) sinusoidal MF and 1.5 mT (25 Hz) pulsed MF, during 40 days of aging, in combination with UVC radiation (50 J/m^2^) and/or thermal shock (52°C). Cell survival was evaluated by clonogenic assay. The exposure of yeast to pulsed MF produces an acceleration of aging, which is not observed in cells exposed to sinusoidal MF. The pulsed MF modifies the cellular response to damaging agents only in aged *S. cerevisiae* cells. In this sense, the pulsed MF applied increases the damage induced by UVC radiation and by thermal shock. In contrast, the sinusoidal MF used has no effect.

## Introduction

The study of the effects of the magnetic field (MF) on living matter continues to be a dilemma. Epidemiological and experimental studies have been carried out for several decades. Until now, the interaction mechanisms of MF with living matter that explain the observed phenomena are unknown (García-Minguillán et al. 2020). Despite this, they are considered potentially carcinogenic (IARC Working Group on the Evaluation of Carcinogenic Risks to Humans 2010).

There are two types of studies published, those in which the damage is induced directly by the MF and those in which the MF, without causing a direct damage, interacts with other agents (physical or chemical) increasing the damage induced by them. In relation to this type of studies, an increase in the damage produced by agents such as SnCl_2_, FeCl_2_, H_2_O_2_, N-methyl-N0-nitro-N-nitrosoguanidine (MNNG), 1,4-benzenediol (BD), 1,2,4-benzenetriol (BT), 4-nitroquinoline N-oxide (4NQO), X-ray, and ultraviolet (UV) radiation; in co-exposure with MF of different magnetic flux density and frequency, on different cells and tissues has been reported (Ruiz-Gómez and Martínez-Morillo 2009).

The results obtained in studies where the damage to DNA by MF is evaluated are in many cases contradictory. It is well known that UV radiation causes DNA damage, inducing the arrest of the yeast cell cycle in the G2 phase. In this sense, Markkanen et al. (2001) described that MF in co-exposure with UV produces a greater delay in the cell cycle, along with a greater recovery time after the induced damage.

In a previously published work we found that the exposure to pulsed MF (1.5mT, 25Hz) alters the cytotoxic activity of cytostatic agents (vincristine, mitomycin C and cisplatin) in a colon carcinoma cell line (Ruiz-Gómez et al. 2002). We subsequently observed that exposure to a pulsed MF in combination with ultraviolet C (UVC) increases the mortality induced by radiation in MCF7 cells of breast cancer (Ruiz-Gómez and Martínez-Morillo 2005). These effects are greater when the MF is applied at the same time as the damaging agent. Recent studies carried out in our laboratory with the same type of MF, but with longer exposure periods (40 days) show that pulsed MF by itself is also capable of producing cellular damage. Specifically, we observed an acceleration of chronological aging in yeasts (Mercado-Sáenz et al. 2019), an increase in the mutation frequency and genomic DNA degradation (Mercado-Sáenz et al. 2022), and an increment in the level of DNA damage induced by methyl methanesulfonate (MMS) and bleomycin (López-Díaz et al. 2022).

To this day, it is still necessary to carry out more laboratory studies that provide new data on observed effects, exposure conditions and possible mechanisms of interaction with living matter to clarify the effects on health. In this regard, there are very few works that evaluate the effect of MF during cellular aging.

The present work aims to study whether low frequency and intensity pulsed (25 Hz) and sinusoidal (50 Hz) MF exposure produce alterations in the cell killing effect of UVC radiation and thermal shock during the chronological aging of *S. cerevisiae*.

## Materials and methods

### Yeast strain and culture medium

The eukaryotic cell model used was the *S. cerevisiae* WS8105-1C (ATCC® 200383™) strain (haploid wild type (wt)). Genotype: *MATalpha, ade2, arg4-17, trp1-289, ura3-52*. Yeast vegetative growth was in Yeast Extract:Peptone:Dextrose (YPD) broth (1:2:2 %) ± 2% Bacto-agar (Mercado-Sáenz et al. 2017). Reagents from Difco, BD & Co. Sparks, MD-USA).

The medium to assay the chronological aging of cells was a synthetic dextrose complete (SDC) (2 % dextrose, 0.5 % ammonium sulphate, 0.17 % yeast nitrogen base, 0.15 % aminoacids mixture, 4X auxotrophic markers [ade, arg, trp, ura]) (Fabrizio and Longo 2007). Reagents from Difco, BD & Co. Sparks, MD-USA).

### Sinusoidal magnetic field exposure system

Two Helmholtz coils (Phywe España, S.A., Madrid-Spain) were used to MF generation (40 cm in diameter, 154 turns of 1.4 mm copper wire). The coil centres were separated 20 cm and the whole system was mounted on a wooden frame. They were connected in series and axial directed. Under this configuration, the magnetic currents flow perpendicular to the coils, therefore parallel to the main axis of the test tubes with yeast in the experimental setup. The homogeneity of the MF was ±3 % at samples location within the coils. Therefore, this was the error measured in the MF spatial distribution. The output of an autotransformer (Saber, S.L., Barcelona-Spain) connected to the electric energy supply was our electric source for sinusoidal 50 Hz alternating current (sinusoidal 50 Hz MF). With the use of an oscilloscope (Hameg HM 204; Hameg Instruments, S.L., Barcelona-Spain) we monitored the waveform generated. The amplitude of the current intensity was measured by an amperimeter (Digital Multimeter MD-200; Promax, S.A., Barcelona-Spain). The magnetic flux density was monitored by a Digital Teslameter (Phywe España, S.A., Madrid-Spain) by the Hall effect using an axial probe (accuracy: ±3 %). All samples (controls and MF-treated) were subjected to the same temporal coincidence of treatment, manipulation and environmental conditions of temperature (23°C) in an air-conditioned room with standard thermometers to monitor the ambient temperature in the lab. The use of a conventional fan facilitated the dissipation of the heat generated in the solenoids. The control samples were placed 5 m away from the coils. The conditions of assay were 2.45 mT, 50 Hz sinusoidal MF, continuously during 40 days.

### Pulsed magnetic field exposure system

To generate a pulsed MF, a Helmholtz type equipment was used whose configuration and characteristics are described in Ruiz-Gómez et al. (2002) and Ruiz-Gómez and Martínez-Morillo (2005). Briefly, the device is made up of two (10.5x15 cm) air core coils, connected in series. The equipment generates rectangular voltage pulses of 25 Hz (Pulsatrón─CEM-84/J, J.&J. Electromédica, Málaga-Spain). The waveform of the applied voltage at 25 Hz was composed of groups of 15 square pulses (1.5 mT peak, 180 μs width, 20 μs gap) (López-Díaz et al. 2022). The samples were exposed to MF 8 hours a day, for 40 days. The equipment used was calibrated by the manufacturer. The magnetic flux density used in the assays was the maximum that the equipment can generate. Other authors, such as Li and Chow (2001) used similar values in their exposures.

### Background magnetic field

The mean value of the DC geomagnetic field in Málaga was 42.95 μT (33.20 μT vertical; 27.225 μT horizontal, 3°5’ Western) (National Geographic Institute, Madrid-Spain). The MF background value in the lab was 0.68 μT (López-Díaz et al. 2014).

### Chronological aging

Chronological aging was evaluated by culture in SDC medium. First, 1.5x10^6^ cells/mL were inoculated in 20 mL of SDC medium. The seeded flasks were cultured until the stationary phase was reached, keeping them at 30°C while shaking at 300 rpm, for 4 days. The chronological aging process began after reaching the early stationary phase. Cultures were transferred to 15 mL tubes, divided into three groups to be placed in the MF exposure equipment and at control location. There was no agitation at this stage. The tubes were vortexed repeatedly each day to prevent sedimentation and keep the yeasts in suspension. The trials lasted 40 days, maintaining stable temperature conditions at 23°C. The control samples were placed 5 meters away from the samples exposed to MF. A fan was used to dissipate the excess of heat produced in the solenoids (Mercado-Sáenz et al. 2019). The surviving fraction was calculated periodically from the data obtained from the clonogenic assays (Fabrizio and Longo 2007).

### Cell density

The cell concentration was measured every day by spectrophotometry at 600nm (OD600) (Helios ε, Unicam, Cambridge, UK). The OD600 values <1.0 are linear with the number of cells for *S. cerevisiae*.

### Clonogenic survival

Cell survival in the control and MF-exposed samples was evaluated by clonogenic assay (quantitative) and drop test (semiquantitative). For semiquantitative assessment of survival, five 10-fold serial dilutions of each sample were prepared and spotted 5 μL of each on YPD plates (Umezu et al. 1998). The quantitative evaluation consisted in spread 100 μL of various serial dilutions of each sample on YPD plates. All plates were incubated for 3 days at 30°C. Afterwards, the number of colonies grown was counted and the surviving fraction was calculated (Franken et al. 2006; Ruiz-Gómez et al. 2008).

### Ultraviolet C radiation exposure

On days 0, 20 and 40 of each experiment during aging, samples were taken from the cell suspensions exposed to MF and controls. Serial dilutions (10^-1^, 10^-2^ and 10^-3^) from an initial culture of 1.5x10^6^ cells/mL at day 0 were made and 100 μL of each dilution was spread out on YPD plates. The plates were then irradiated with ultraviolet C (UVC) radiation, cultured at 30°C for 3-5 days and subsequently the colonies grown were counted. From the data obtained, the surviving fraction was calculated.

A UVC germicidal fluorescent lamp was used (Osram, S.A., Sylvania G30W-T8, Japan) (power: 30 W; UVC: 8 W (253.7 nm); intensity: 0.22 W/m^2^). The energy density applied was 50 J/m^2^. Control cells were irradiated in a simulated manner. The opened plates were placed for irradiation under the lamp, inside a laminar flow cabinet, for 15 seconds at a distance of 22 cm. The dosimetry of the lamp was measured using a UV203 UV radiometer (Macam, Scotland, UK).

### Thermal shock exposure

On days 0, 20 and 40 of chronological aging, samples were taken from yeasts exposed to MF and from non-exposed controls and subsequently they were exposed to thermal shock (52°C).

To perform the thermal shock exposure, a portion of each sample (500 μL) was taken in an Eppendorf tube. The samples were placed in a thermostatic bath with stirring at 52°C and maintained for 15 minutes; both those exposed to MF and controls. Then, to stop the thermal shock process, the samples were cooled to 4°C, by introducing the Eppendorf tubes in a rack containing ice water. Temperature was controlled with a conventional thermometer.

Once the samples were cooled, a clonogenic assay was performed. After a culture period of 3-5 days at 30°C, the grown colonies were counted and the surviving fraction was calculated. Samples exposed to cold (4°C) but not to thermal shock were used as controls.

### Statistical analyses

Statistical analyses were performed using the Analysis of Variance (ANOVA) and the Student's *t*-test. A value of *p*<.05 was considered significant. The assays were performed in triplicate, repeating each one 3 times (*n*=8–16).

## Results

### Effect of magnetic fields on aging

**Fig. 1:**
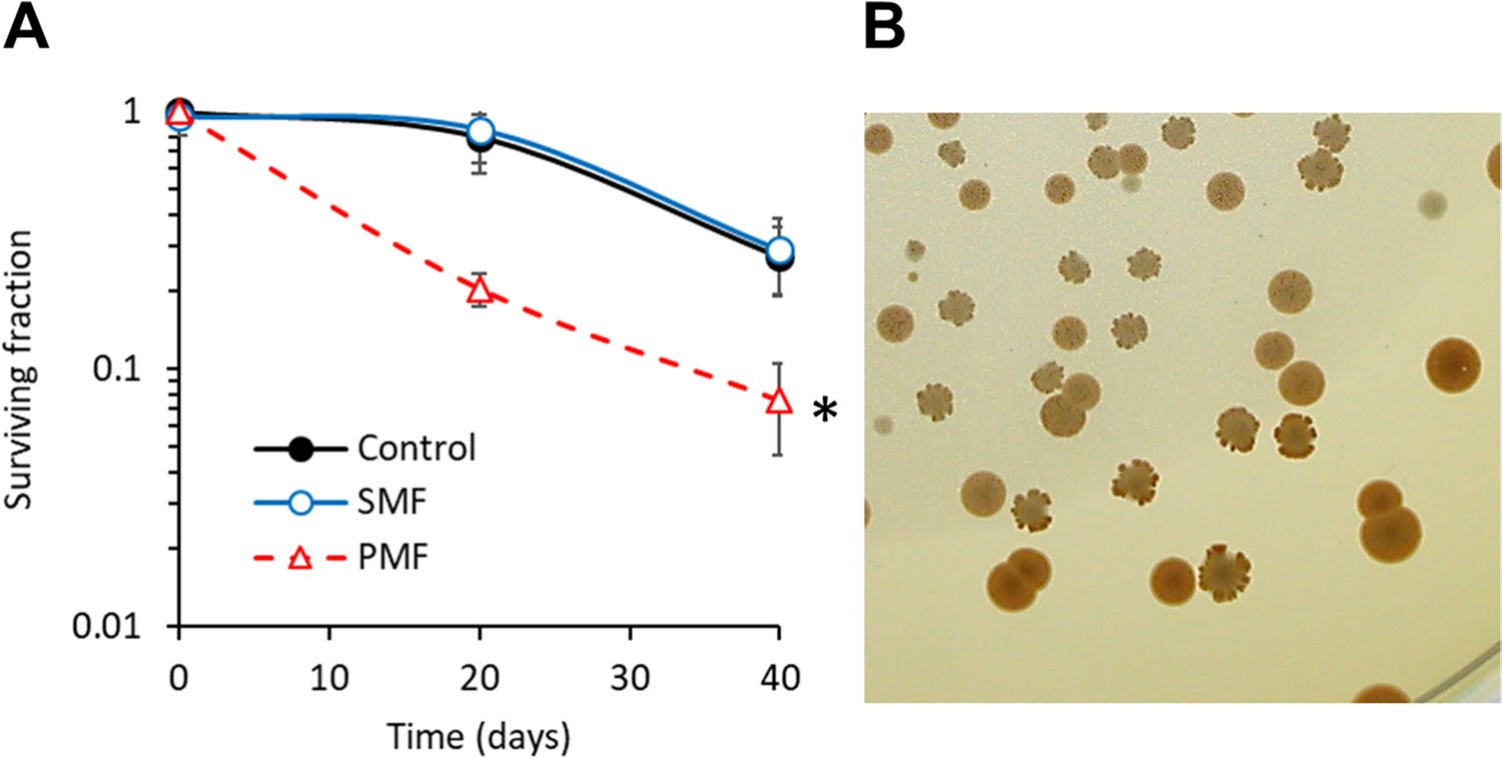
Chronological aging. **A**) Effect of sinusoidal (2.45mT, 50Hz) and pulsed (1.5mT, 25Hz) magnetic field exposures on yeast aging. **B**) Lobed colonies indicative of chronological aging. Mean ± SD. *p<0.001 ANOVA. SMF: Sinusoidal magnetic field. PMF: Pulsed magnetic field

Figure 1 shows the effect of sinusoidal MF and pulsed MF on chronological aging of yeast. The value of survival decreased with time for all samples. In this way, sinusoidal MF did not show significant differences in relation to control samples (*p*>0.05 ANOVA). However, pulsed MF showed bigger and significant differences in relation to controls (*p*<0.001 ANOVA) (Fig. 1A). Surviving fractions were 0.21 and 0.08 for pulsed MF samples on day 20 and 40 respectively, in relation to the values of 0.80 and 0.27 found for control samples. Therefore, pulsed MF induced an acceleration of aging. Figure 1B shows the typical lobed colonies indicatives of the aging phenomenon.

### Effect of ultraviolet C radiation on yeasts exposed to magnetic fields during aging

In order to evaluate if sinusoidal and pulsed MF produce alterations in the cellular mortality induced by UVC radiation, yeast samples were exposed to UVC during chronological aging at days 0, 20 and 40 of aging.

**Fig. 2:**
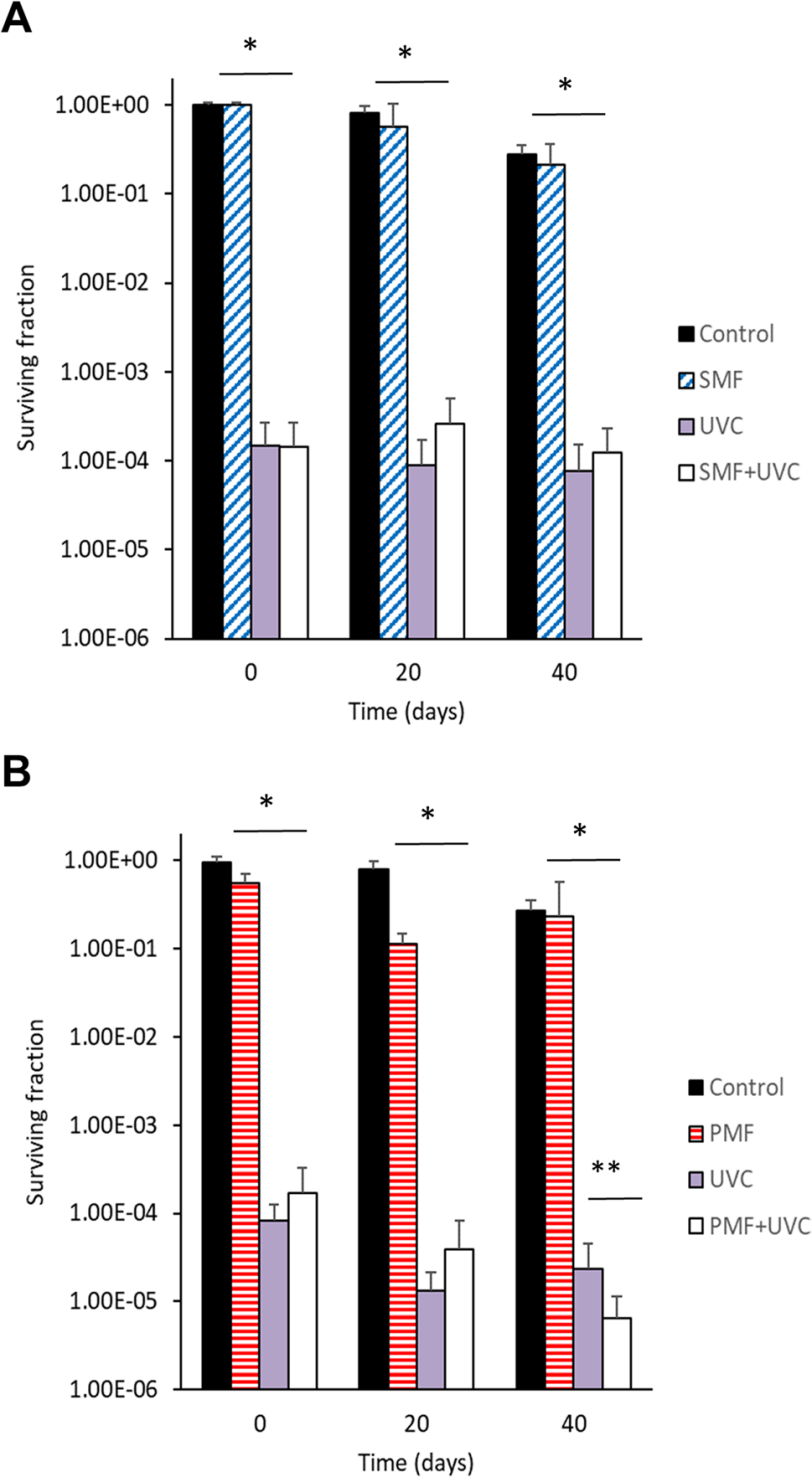
Effect of magnetic field and ultraviolet C radiation on the chronological aging of yeast. The energy density applied was 50 J/m^2^. **A**) Sinusoidal magnetic field exposure (2.45mT, 50Hz). **B**) Pulsed magnetic field exposure (1.5mT, 25Hz). Mean ± SD. *p<0.001, **p<0.05; Student *t*-test. SMF: Sinusoidal magnetic field. PMF: Pulsed magnetic field. UVC: ultraviolet C

Figure 2A shows that the cytotoxic effect of UVC radiation caused a decrease in cell survival, reaching on days 0, 20 and 40 a surviving fraction value of 1.46x10^-4^, 8.95x10^-5^ and 7.59x10^-5^, respectively. The differences in relation to the untreated controls were statistically significant (*p*<0.001, Student *t*-test).

The surviving fraction obtained on days 0, 20 and 40 of aging for the groups treated with sinusoidal MF+UVC were similar to the groups exposed only to UVC. In this sense, the differences were statistically significant in relation to the control groups (*p*<0.001, Student *t*-test) but not significant with respect to the groups treated only with UVC (*p*>0.05, Student *t*-test) (Fig. 2A).

The groups treated only with sinusoidal MF also showed no statistically significant differences in relation to controls, during the aging process evaluated on days 0, 20 and 40 (*p*>0.05, ANOVA).

The data indicate that exposure to sinusoidal MF during aging did not modify the cellular response to UVC radiation.

Subsequently, the effect of UVC radiation in combination with pulsed MF exposure was evaluated. The cytotoxic effect of UVC radiation can be observed clearly in Fig. 2B, which caused a significant decrease in cell survival. When pulsed MF exposure was combined with UVC, the cytotoxic effect increased obtaining a surviving fraction value of 6.51x10^-6^ at day 40 of aging in relation to the value of its matched control (2.37x10^-5^) treated only with UVC (*p*<0.05, Student *t*-test). Therefore, the cytotoxic effect of UVC radiation combined with pulsed MF was 3.63 times higher compared to the group treated only with UVC.

There were no statistically significant differences between the groups treated with pulsed MF+UVC in relation to those treated only with UVC, for days 0 and 20 of aging (*p*>0.05, ANOVA, Student *t*-test).

The results obtained suggest that chronological aging was altered by exposure to pulsed MF. In addition, they indicate that exposure to pulsed MF increases the damage induced by UVC radiation only in aged cells.

### Effect of exposure to thermal shock in yeasts exposed to magnetic fields during aging

In order to evaluate whether sinusoidal and pulsed MF produce alterations in the cellular mortality induced by exposure to thermal shock, yeast samples were exposed to 52°C for 15 minutes, during the chronological aging of cells at days 0, 20 and 40 of aging.

**Fig. 3:**
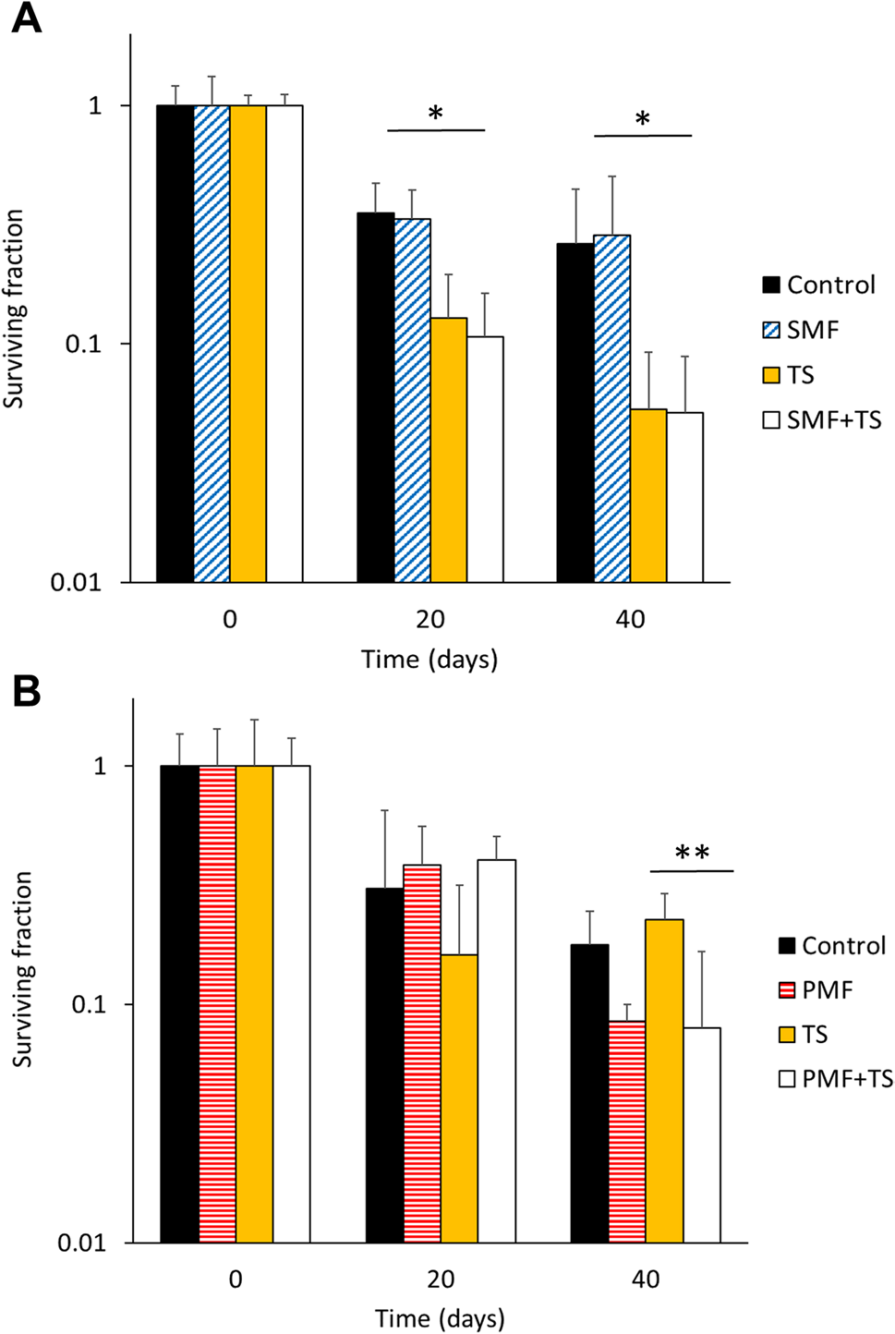
Effect of magnetic field and thermal shock (52°C, 15 min.) on the chronological aging of yeast. **A**) Sinusoidal magnetic field exposure (2.45mT, 50Hz). **B**) Pulsed magnetic field exposure (1.5mT, 25Hz). Mean ± SD. *p<0.005, **p<0.001; Student *t*-test. SMF: Sinusoidal magnetic field. PMF: Pulsed magnetic field. TS: Thermal shock

Figure 3 shows the effect of thermal shock as a function of exposure time and chronological aging. In the intermediate and final stages of aging (days 20 and 40) exposure to thermal shock produced a higher mortality, which increased with aging.

The groups subjected to sinusoidal MF showed a behavior similar to the control groups during aging, with no statistically significant differences (*p*>0.05, Student *t*-test). However, samples treated with thermal shock showed a decrease in surviving fraction compared to controls, being the differences statistically significant (*p*<0.005, Student *t*-test) (Fig. 3A). The combination of thermal shock and sinusoidal MF did not produce survival alterations (*p*>0.05, Student *t*-test). The results obtained suggest that exposure to sinusoidal MF during aging does not modify the cellular response to thermal shock exposure.

Subsequently, the effect of pulsed MF combined with thermal shock was evaluated. Samples exposed to pulsed MF showed less surviving fraction than their matched controls due to the influence of this type of fields along the aging process (Fig. 1A). The normalized data in relation to their respective matched controls showed that the aged yeasts (day 40) exposed to a combination of pulsed MF and thermal shock presented a lower surviving fraction value in relation to that obtained for the group treated only with heat shock. The differences were statistically significant (*p*<0.001, Student *t*-test) (Fig. 3B).

The results obtained suggest that exposure to pulsed MF increases the damage induced by thermal shock only in aged cells.

## Discussion

It is surprising today that there is still a dilemma about the effects of low intensity and low frequency electromagnetic fields on living organisms, despite the large amount of published literature. This circumstance is due, on the one hand, to the large number of contradictory studies and, on the other, to the difficulty of replicating experiments in different laboratories. This difficulty lies mainly in the large number of variables to be controlled in laboratory tests, both biological and physical; not counting the fact that there may be other variables that we do not know about. In this way, the results obtained by other authors are based on different cell lines of different origin and biological status. Also, different equipments, exposure protocols (in relation to time, frequency, waveform, magnetic flux density, field homogeneity) and manipulation contribute to the diversity of results.

Although there are numerous studies on MF, UVC radiation and thermal shock effects, it is difficult to find identical laboratory protocols and therefore it is difficult to compare results. On the contrary, there are practically no studies that investigate the effect of MF on aging.

The goodness of an experiment is based not only on the results obtained, but also on the principles of repeating them, for which adequate information is needed on the conditions in which the experiment is carried out (McCann et al. 1998). It is common to find MF articles in which the authors have forgotten to include some aspects related to exposure conditions, such as field homogeneity, wave type, and even temperature control.

MF exposure generates heat in the solenoid or coils due to the passage of electrical current, which produces an increase in the temperature that can heat the samples. This makes it necessary to control the temperature of both the exposed samples and the control in order to minimize the difference between them so that the variations that could be found are not simply due to a thermal effect (Ruiz-Gómez et al. 2008).

Electromagnetic fields are non-ionizing radiation. In this sense, they do not have enough energy to produce ionizations in atoms. However, they can affect cell homeostasis and influence processes related to cell proliferation through their influence on the free radicals generated, which could affect the carcinogenic process. In this regard, it is important to highlight that the exposure conditions used in the experimental studies are higher in intensity than the values found in the environment and therefore are far from the maximum exposure limits established by international regulatory bodies.

Currently, the scientific and clinical evidence of a relationship between exposure to certain levels of MF and the risk of cancer is very low. This circumstance adds to the lack of accurate data regarding dose-response relationships and interaction mechanisms (Kavet 1996; Moulder and Foster 1995; Foster et al. 1997; McCann et al. 1998; De la Peña et al. 2002).

We live exposed to a natural MF, so it is logical to think that any living being has the capacity of adaptation to another MF of similar characteristics, such as continuous artificial fields. However, adaptation to pulsed or even discontinuous MF would be more complex. This may be a feasible explanation of why there is a response or effect against the pulsed field used and not the continuous sinusoidal one. The adaptation effect would be even more complex if several physical agents are combined, such as thermal shock and UVC radiation (Pasquinelli et al. 1993).

In previous studies conducted in our laboratory, it was concluded that pulsed MF (1 Hz and 25 Hz, 1.5 mT) can induce alterations of the effect of cytostatic agents on a human colon adenocarcinoma cell line, producing a synergistic effect which increases the response when the field is applied simultaneously with pharmacological therapy (Ruiz-Gómez et al., 2002). It was also observed that pulsed MF exposures (25 Hz, 0.75 mT) in combination with UVC (253.7 nm) have the ability to increase the effects of UVC radiation on MCF-7 breast cancer cells (Ruiz-Gómez and Martínez-Morillo 2005). In addition, no alteration in the growth of *S. cerevisiae* was found after exposure to 50 Hz static and sinusoidal MF (0.35 and 2.45 mT) (Ruiz-Gómez et al. 2004). Recent reports indicate that pulsed MF (25 Hz, 1.5 mT) has the ability to accelerates the aging process (Mercado-Sáenz et al. 2019), alters the damage induced by methyl methanesulfonate and bleomycin (López-Díaz et al. 2022) and produces genomic DNA damage during the chronological aging of yeast cells (Mercado-Saénz et al. 2022).

In our study, we found that although the sinusoidal MF applied did not produce alterations in the chronological aging of yeasts, the pulsed field did affect cell aging; significantly reducing the surviving fraction, despite the fact that the time of exposure to pulsed MF was less than the sinusoidal MF exposure time. These results agree with those published by other authors in *C. elegans*, which indicate a significant decrease in life expectancy in nematodes exposed to MF (Hung et al. 2010).

It has been reported that non-ionizing radiation of extremely low frequency, as is the case with MF of 50 Hz, affect a large number of biochemical processes, such as the synthesis of nucleic acids and proteins, the production of hormones, the immune response, the increase in DNA breakage, cell growth and differentiation, enzymatic activity, permeability and transport through membranes and increase free radicals production; conditioning the appearance of cancer (Wei and Wang 2022). Recently, García-Minguillán et al. (2020) reported a cell viability decrease with a strong frequency dependence after exposure of cells to a 30 μT and 100 μT during 24 h and 7 days, respectively.

It has also been found that although the sinusoidal MF does not seem to cause a break in the DNA chain, in the case of the pulsed MF a greater degradation of the DNA is observed; therefore, our results coincide with those obtained by other authors who observed induction of DNA double strand breaks (Dicarlo et al. 1999, Scarfi et al. 2005). It should be noted that there seems to be a dose-response relationship between MF strength and DNA strand breakage (Invancsits et al. 2003), although these results have not been corroborated by studies carried out in independent laboratories (Scarfi et al. 2005).

Since electric fields cannot penetrate the organism, it is widely accepted that any biological effect induced by artificial electromagnetic fields must be due to the magnetic component of the field, or to the electric fields and currents that these MF induce in the organism (Moulder and Foster 1995).

To study the effects of UVC radiation, the cells were irradiated with an energy density of 50 J/m^2^. A similar dose of UVC was previously used by Dicarlo et al. (1999) (30 J/m^2^ and 45 J/m^2^), Wang et al. (1999) (20 J/m^2^), and Ruiz-Gómez and Martínez-Morillo (2005) (6-59 J/m^2^). Under the study conditions used, it should be noted that the cytotoxic effect of UVC radiation is increased when the exposure is combined with the pulsed MF, which may indicate that the pulsed field could act as a radio-sensitizing agent (Ruiz-Gómez and Martínez-Morillo 2005). This phenomenon has been previously described by Nindl et al. (2002) who published a synergistic effect between UV radiation and pulsed MF.

UVC radiation (240-290 nm) firstly produces DNA damage and secondly induces the formation of reactive oxygen species (ROS), which interact damaging multiple molecules (Dicarlo et al., 1999). In this sense, Roy et al. (1995) showed a relationship between exposure to MF and ROS evolution in the presence of oxidative chemical agents; producing a significant increase in free radicals after the application of MF. Dicarlo et al. (1999) reported that MF exposure (8 μT, 60 Hz, 20 minutes) induces protection against damage subsequently generated by UVC radiation. These authors hypothesized that the MF applied can stimulate cell repair processes such as the induction of ROS-mediated heat shock proteins (HSP); so the antioxidant system in the cells is increased. They also suggest that exposures to MF can be beneficial at low doses.

Our results demonstrate that sinusoidal and pulsed MF did not modify the cellular response to thermal shock during aging. However, other authors find evidence of cells protection against external aggressions by thermal shock exposure (Dicarlo et al. 1999). Fabrizio et al. (2001) concludes that there seems to be a relationship between life expectancy and resistance to stress. The effect is higher when the exposure time to thermal shock is increased.

In situations of thermal stress, the organisms elaborate a thermal shock response that includes the synthesis of HSPs, responsible for the correct folding of other proteins, the recovery of anomalous proteins, and finally the prevention of the aggregation of unfolded proteins and their elimination (Hartl 2016; Peinado-Ruiz et al. 2022). HSPs play an important role not only in cytoprotection processes against the damaging effects of different stressors, but also in cellular and tissue repair. All cells, both eukaryotic and prokaryotic, react to hyperthermia by the thermal shock response that results in the activation of genes that code for a group of stress-activated proteins (Kyriakou et al. 2022; Ahmed et al. 2020). HSPs are also involved in the processes of protection against oxidative stress caused by UV radiation, or situations that involve a deficit in antioxidant systems (López-Diazguerrero et al. 2013). In addition, it is important to consider that HSPs (HSP27, HSC70, HSP70, HSF1 and HSP90) expression is altered in normal and tumor cells exposed to different stressors (pharmacological, hypoxic, thermal and oxidative). These alterations induce cell resistance to physical and chemical agents (Peinado-Ruiz et al. 2022).

The mitochondrial theory points out that free radicals easily damage mitochondria, being responsible for aging (Wiley, 2007). Other studies corroborate that changes in the mitochondrial genome have a great influence on the longevity of individuals (Zhang et al. 2003). According to the theory of mutation, mutations accelerate the aging process (Warner et al. 1987). The theory of free radicals indicates that the antioxidative defense, that reduce the damage of ROS, contributes to counteract the cellular aging (Yokozawa et al. 2004). This theory is compatible with experimental observations of mitochondrial aging. The generation of energy at cellular level induces the formation of free radicals. By this reason, mitochondria is an easy target to be damage by ROS. On the other hand, free radicals have paramagnetic properties and play an important role in cellular aging (Birren 2006; Wiley 2007).

In the mitochondria, organelles with scarce machinery that repairs damaged DNA, the basic processes of oxidative metabolism and ROS production are developed. The genetic alterations of nuclear genes that encode mitochondrial enzymes, especially antioxidants, and mitochondrial genes that totally or partially carry the coding information of some components of the respiratory chain, deserve special attention. In addition, the exclusively maternal transmission of mtDNA could be related to the relative weight of paternal and maternal inheritances in the phenomenon of aging (Allen 1996).

While the unfinished nature of the investigation of the biological effects of MF continues, it is risky to take definitive measures in this regard, aimed at mitigating the possible effects. The most sensible policies suggest that while there is a pending of scientific conclusive answers, reasonable measures must be taken; that is, with the minimum inconveniences, both economic and technical, to reduce exposure to low frequency fields.

Public concern is maintained based on the contradictory information disseminated by the media and the inability of scientists to ensure that there is no risk.

## Conclusion

The exposure of yeast to pulsed MF produces an acceleration of aging, which is not observed in cells exposed to sinusoidal MF. The pulsed MF modifies the cellular response to damaging agents only in aged *S. cerevisiae* cells. In this sense, the pulsed MF applied (1.5mT, 25Hz) increases the damage induced by ultraviolet C radiation (50 J/m^2^) and by thermal shock (52°C, 15 min.). In contrast, the sinusoidal MF used (2.45mT, 50Hz) has no effect.

## Declarations

### Data Availability Statement

All data included in this study are available upon reasonable request by contacting the corresponding author.

## References

[CR1] Ahmed K, Zaidi SF, Mati-Ur-Rehman RR, Kondo T (2020). Hyperthermia and protein homeostasis: Cytoprotection and cell death. J Therm Biol.

[CR2] Allen JF (1996). Separate sexes and the mitochondrial theory of ageing. J Theor Biol.

[CR3] Birren JE (2006). Encyclopedia of gerontology.

[CR4] De la Peña FL, Pastor Vega JM, Ruiz Gómez MJ, Martínez MM (2002). Riesgo laboral y residencial por exposición a campos electromagnéticos. Mapfre Med.

[CR5] Dicarlo AL, Hargis MT, Penafiel LM, Litovitz TA (1999). Short-term magnetic field exposures (60 Hz) induce protection against ultraviolet radiation damage. Int J Radiat Biol.

[CR6] Fabrizio P, Longo VD (2007). The chronological life span of Saccharomyces cerevisiae. Methods Mol Biol.

[CR7] Fabrizio P, Pozza F, Pletcher SD, Gendron CM, Longo VD (2001). Regulation of longevity and stress resistance by Sch9 in yeast. Science..

[CR8] Foster KR, Erdreich LS, Moulder JE (1997). Weak electromagnetic fields and cancer in the context of risk assessment. Proc IEEE.

[CR9] Franken NA, Rodermond HM, Stap J, Haveman J, van Bree C (2006). Clonogenic assay of cells in vitro. Nat Protoc.

[CR10] García-Minguillán O, Prous R, Ramirez-Castillejo MDC, Maestú C (2020). CT2A Cell Viability Modulated by Electromagnetic Fields at Extremely Low Frequency under No Thermal Effects. Int J Mol Sci.

[CR11] Hartl FU (2016). Cellular Homeostasis and Aging. Annu Rev Biochem.

[CR12] Hung YC, Lee JH, Chen HM, Huang GS (2010). Effects of static magnetic fields on the development and aging of *Caenorhabditis elegans*. J Exp Biol.

[CR13] IARC Working Group on the Evaluation of Carcinogenic Risks to Humans (2010). IARC monographs on the evaluation of carcinogenic risks to humans. Ingested nitrate and nitrite, and cyanobacterial peptide toxins. IARC monographs on the evaluation of carcinogenic risks to humans / World Health Organization.

[CR14] Ivancsits S, Diem E, Jahn O, Rüdiger HW (2003). Intermittent extremely low frequency electromagnetic fields cause DNA damage in a dose-dependent way. Int Arch Occup Environ Health.

[CR15] Kavet R (1996) EMF and current cancer concepts. Bioelectromagnetics. 17(5):339–357 10.1002/(SICI)1521-186X(1996)17:5<339::AID-BEM1>3.0.CO;2-4 10.1002/(SICI)1521-186X(1996)17:5<339::AID-BEM1>3.0.CO;2-4 8915543

[CR16] Kyriakou E, Taouktsi E, Syntichaki P (2022). The Thermal Stress Coping Network of the Nematode Caenorhabditis elegans. Int J Mol Sci.

[CR17] Li SH, Chow KC (2001). Magnetic field exposure induces DNA degradation. Biochem Biophys Res Commun.

[CR18] López-Díaz B, Mercado-Sáenz S, Burgos-Molina AM, González-Vidal A, Sendra-Portero F, Ruiz-Gómez MJ (2022). Genomic DNA damage induced by co-exposure to DNA damaging agents and pulsed magnetic field. Int J Radiat Biol Sep.

[CR19] López-Díaz B, Mercado-Sáenz S, Martínez-Morillo M, Sendra-Portero F, Ruiz-Gómez MJ (2014). Long-term exposure to a pulsed magnetic field (1.5 mT, 25 Hz) increases genomic DNA spontaneous degradation. Electromagn Biol Med.

[CR20] López-Diazguerrero NE, González Puertos VY, Hernández-Bautista RJ, Alarcón-Aguilar A, Luna-López A, Königsberg FM (2013). Hormesis: lo que no mata, fortalece [Hormesis: What doesn't kill you makes you stronger]. Gac Med Mex.

[CR21] Markkanen A, Juutilainen J, Lang S, Pelkonen J, Rytömaa T, Naarala J (2001). Effects of 50 Hz magnetic field on cell cycle kinetics and the colony forming ability of budding yeast exposed to ultraviolet radiation. Bioelectromagnetics.

[CR22] McCann J, Dietrich F, Rafferty C (1998). The genotoxic potential of electric and magnetic fields: an update. Mutat Res.

[CR23] Mercado-Sáenz S, Burgos-Molina AM, López-Díaz B, Sendra-Portero F, Ruiz-Gómez MJ (2019). Effect of sinusoidal and pulsed magnetic field exposure on the chronological aging and cellular stability of S. cerevisiae. Int J Radiat Biol.

[CR24] Mercado-Sáenz S, López-Díaz B, Burgos-Molina AM, Sendra-Portero F, González-Vidal A, Ruiz-Gómez MJ (2022). Exposure of S. cerevisiae to pulsed magnetic field during chronological aging could induce genomic DNA damage. Int J Environ Health Res.

[CR25] Mercado-Saenz S, Lopez-Diaz B, Sendra-Portero F, Martinez-Morillo M, Ruiz-Gomez MJ (2017). Inactivation of RAD52 and HDF1 DNA repair genes leads to premature chronological aging and cellular instability. J Biosci.

[CR26] Moulder JE, Foster KR (1995). Biological effects of power-frequency fields as they relate to carcinogenesis. Proc Soc Exp Biol Med.

[CR27] Nindl G, Hughes EF, Johnson MT, Spandau DF, Vesper DN, Balcavage WX (2002). Effect of ultraviolet B radiation and 100 Hz electromagnetic fields on proliferation and DNA synthesis of Jurkat cells. Bioelectromagnetics..

[CR28] Pasquinelli P, Petrini M, Mattii L, Galimberti S, Saviozzi M, Malvaldi G (1993). Biological effects of PEMF (pulsing electromagnetic field): an attempt to modify cell resistance to anticancer agents. J Environ Pathol Toxicol Oncol.

[CR29] Peinado-Ruiz IC, Burgos-Molina AM, Sendra-Portero F, Ruiz-Gómez MJ (2022). Relationship between heat shock proteins and cellular resistance to drugs and ageing. Exp Gerontol.

[CR30] Roy S, Noda Y, Eckert V, Traber MG, Mori A, Liburdy R, Packer L (1995). The phorbol 12-myristate 13-acetate (PMA)-induced oxidative burst in rat peritoneal neutrophils is increased by a 0.1 mT (60 Hz) magnetic field. FEBS Lett.

[CR31] Ruiz-Gómez MJ, de la Peña L, Prieto-Barcia MI, Pastor JM, Gil L, Martínez-Morillo M (2002). Influence of 1 and 25 Hz, 1.5 mT magnetic fields on antitumor drug potency in a human adenocarcinoma cell line. Bioelectromagnetics.

[CR32] Ruiz-Gómez MJ, Martínez-Morillo M (2005). Enhancement of the cell-killing effect of ultraviolet-C radiation by short-term exposure to a pulsed magnetic field. Int J Radiat Biol.

[CR33] Ruiz-Gómez MJ, Martínez-Morillo M (2009). Electromagnetic fields and the induction of DNA strand breaks. Electromagn Biol Med.

[CR34] Ruiz-Gómez MJ, Merino-Moyano MD, Cebrián-Martín MG, Prieto-Barcia MI, Martínez-Morillo M (2008). No effect of 50 Hz 2.45 mT magnetic field on the potency of cisplatin, mitomycin C, and methotrexate in S. cerevisiae. Electromagn Biol Med.

[CR35] Ruiz-Gómez MJ, Prieto-Barcia MI, Ristori-Bogajo E, Martínez-Morillo M (2004). Static and 50 Hz magnetic fields of 0.35 and 2.45 mT have no effect on the growth of *Saccharomyces cerevisiae*. Bioelectrochemistry.

[CR36] Scarfí MR, Sannino A, Perrotta A, Sarti M, Mesirca P, Bersani F (2005). Evaluation of genotoxic effects in human fibroblasts after intermittent exposure to 50 Hz electromagnetic fields: a confirmatory study. Radiat Res.

[CR37] Umezu K, Sugawara N, Chen C, Haber JE, Kolodner RD (1998). Genetic analysis of yeast RPA1 reveals its multiple functions in DNA metabolism. Genetics.

[CR38] Wang JA, Fan S, Yuan RQ, Ma YX, Meng Q, Goldberg ID, Rosen EM (1999). Ultraviolet radiation down-regulates expression of the cell-cycle inhibitor p21WAF1/CIP1 in human cancer cells independently of p53. Int J Radiat Biol.

[CR39] Warner HR, Butler RN, Richard L, Sprott PD (1987). Modern biological theories of aging.

[CR40] Wei Y, Wang X (2022). Biological effects of rotating magnetic field: A review from 1969 to 2021. Prog Biophys Mol Biol.

[CR41] Wiley J (2007). Encyclopedia of life sciences.

[CR42] Yokozawa T, Satoh A, Cho EJ (2004). Ginsenoside-Rd attenuates oxidative damage related to aging in senescence-accelerated mice. J Pharm Pharmacol.

[CR43] Zhang QM, Tokiwa M, Doi T, Nakahara T, Chang PW, Nakamura N, Hori M, Miyakoshi J, Yonei S (2003). Strong static magnetic field and the induction of mutations through elevated production of reactive oxygen species in Escherichia coli soxR. Int J Radiat Biol.

